# Negative regulation of Bmi-1 by AMPK and implication in cancer progression

**DOI:** 10.18632/oncotarget.6748

**Published:** 2015-12-23

**Authors:** Deqiang Huang, Xiaoling He, Junrong Zou, Pei Guo, Shanshan Jiang, Nonghua Lv, Yuriy Alekseyev, Lingyu Luo, Zhijun Luo

**Affiliations:** ^1^ Research Institute of Digestive Diseases, The First Affiliated Hospital of Nanchang University, Nanchang, Jiangxi, China; ^2^ Graduate Program, Jiangxi Medical College, Nanchang University, Nanchang, Jiangxi, P.R. China; ^3^ Institute of Basic Medical Sciences, Nanchang University, Nanchang, Jiangxi, P.R. China; ^4^ Departments of Pathology and Laboratory Medicine, Boston University School of Medicine, Boston, MA 02118, USA; ^5^ Department of Biochemistry, Boston University School of Medicine, Boston, MA 02118, USA

**Keywords:** AMPK, Bmi-1, LITAF, cancer progression, miRNA

## Abstract

Bmi-1 is a transcriptional regulator that promotes tumor cell self-renewal and epithelial to mesenchymal transition and its upregulation is associated with tumor progression, AMPK is an intracellular fuel-sensing enzyme and plays important roles in tumor cell growth and progression. Thus, the present study aims to examine the regulation of Bmi-1 by AMPK. First, our data revealed that, as compared to adjacent normal tissue, Bmi-1 was highly expressed in gastric cancer, whereas phosphorylation of AMPK (p-AMPK) was reduced. Similar findings were observed in lung adenocarcinomas and appeared that the expression of Bmi-1 was correlated with pathological grades of the cancer, where opposite changes were found in p-AMPK. Second, Metformin, a pharmacological AMPK activator and anti-diabetic drug, or ectopic expression of LKB1, diminished expression of Bmi-1 in cancer cells, an event that was reversed by silencing LKB1. Third, knockdown of LITAF, previously identified as a downstream target of AMPK, upregulated Bmi-1, associated with increased cell viability, colony formation, and migration of cancer cells *in vitro*. Fourth, metformin increased the abundance of miR-15a, miR-128, miR-192, and miR-194, which was prevented by knockdown of LITAF. Accordingly, transfection of these individual miRNAs downregulated Bmi-1. Altogether, our data for the first time suggest a regulatory axis in cancer cells: AMPK upregulates LITAF, which in turn increases miRNAs, leading to attenuation of Bmi-1 expression.

## INTRODUCTION

B-lymphoma Moloney murine leukemia virus insertion region-1 (Bmi-1), a member of the Polycomb transcription repressors, participates in various biological processes, including embryonic development, organ formation, tumorigenesis, and stem cell self-renewal and differentiation [1]. Previous studies have shown that Bmi-1 protein is overexpressed in different types of human cancers, such as lung [2], gastric [3], and breast cancers [4] and leukemia [5, 6]. Thus, Bmi-1 is accepted as an oncogene that alters cell cycle, senescence, and apoptosis by promoting tumor cell self-renewal and epithelial to mesenchymal transition (EMT) [5]. At the transcription level, Bmi-1 cooperates with c-myc to repress expression of tumor suppressor genes including p16Ink4a and p19Arf, thereby preventing apoptosis and stimulating cell proliferation [7]. By contrary, knockdown of Bmi-1 results in cell cycle arrest and inhibition of cell proliferation, but increased cell differentiation [8]. Recent studies have shown that multiple microRNAs could repress translation of *Bmi-1* and thus block proliferation and metastasis of tumor cells [9–13].

Adenosine 5′-monophosphate (AMP)-activated protein kinase (AMPK) is an energy sensor and plays an important role in cellular metabolism and biosynthesis of macromolecules. AMPK is an important effector of the tumor suppressor LKB1. Thus, a large number of studies have shown that activation of AMPK by pharmacological activators such as metformin, 5-amino-1-β-D-ribofuranosyl-imidazole-4-carboxamide (AICAR) and salicylate lead to inhibition of cancer cell proliferation or induce apoptosis [14]. In animal studies, AMPK activation has been shown to inhibit tumorigenesis. Several previous studies have reported that AMPK is reduced in human cancer specimens, suggesting a role in tumorigenesis and tumor progression [15]. Indeed, *in vitro* studies have shown that activation of AMPK activity by pharmacological activators sensitizes cancer cells to chemotherapy [16].

Lipopolysaccharide-induced TNFα factor (LITAF) appears to be a multifunctional small protein consisting of 161 amino acids [17]. It has been characterized as a transcription factor for inflammatory cytokines in macrophages [18]. In response to LPS, LITAF translocates into the nucleus and binds to a specific element on promoters for proinflammatory cytokines such as the TNFα promoter, where it interacts and cooperates with STAT6(B) to activate their transcription [19]. Interestingly, the sequence of LITAF is identical to the Small Integral Membrane Protein of the Lysosome/late Endosome (SIMPLE). Mutations of LITAF/SIMPLE are associated with a genetic disease called Charcot-Marie-Tooth disease type 1C (CMT1C), characterized by demyelinating disorders of peripheral nervous system [20–22]. The precise role for the mutated LITAF in the pathogenesis of this genetic disease remains enigmatic. It has been suggested that the mutants fail to target membrane proteins for recycling and lysosomal degradation, leading to the death of Schwann cells. Another aspect of LITAF function is related to its effect on cancer cells. We have recently identified LITAF as a downstream target of AMPK [23]. The expression of LITAF in prostate cancer cells is upregulated by activation of AMPK and suppressed by a dominant negative mutant of AMPKα1 subunit or its shRNA. Furthermore, silencing of LITAF in prostate cancer cells promotes proliferation, anchorage-independent growth, and xenograft tumor development. Additionally, we found that LITAF participates in transcriptional regulation of TNFSF15, a pro-inflammatory cytokine and also a potent inhibitor of tumor angiogenesis [23]. In line with this, recent studies have documented that expression of LITAF promotes apoptosis and differentiation of acute myeloid leukemia cells [24] and that autophagy is suppressed in lymphoma cells where LITAF was silenced by BCL6 [25].

In the present study, we attempted to examine if AMPK regulates expression of Bmil-1 and explore the underlying mechanisms. We found that expression of Bmi-1 was increased whereas phospho-AMPK was decreased in gastric cancer and lung adenocarcinoma specimens. In cancer cells, we found that metformin activated AMPK, concurrently with upregulation of LIFAF and downregulation of Bmi-1. Interestingly, our data showed that LITAF mediated the effect of metformin on upregulation of miR-15a, miR-128, miR-192, and miR-194, all of which suppressed expression of bmi-1. Altogether, our data for the first time depicted a regulatory axis sequentially tethering AMPK-LITAF-miRNAs-Bmi-1 in cancer cells.

## RESULTS

### Altered expression of Bmi-1 and p-AMPK in gastric cancer tissues and lung cancer tissue

**Figure 1:**
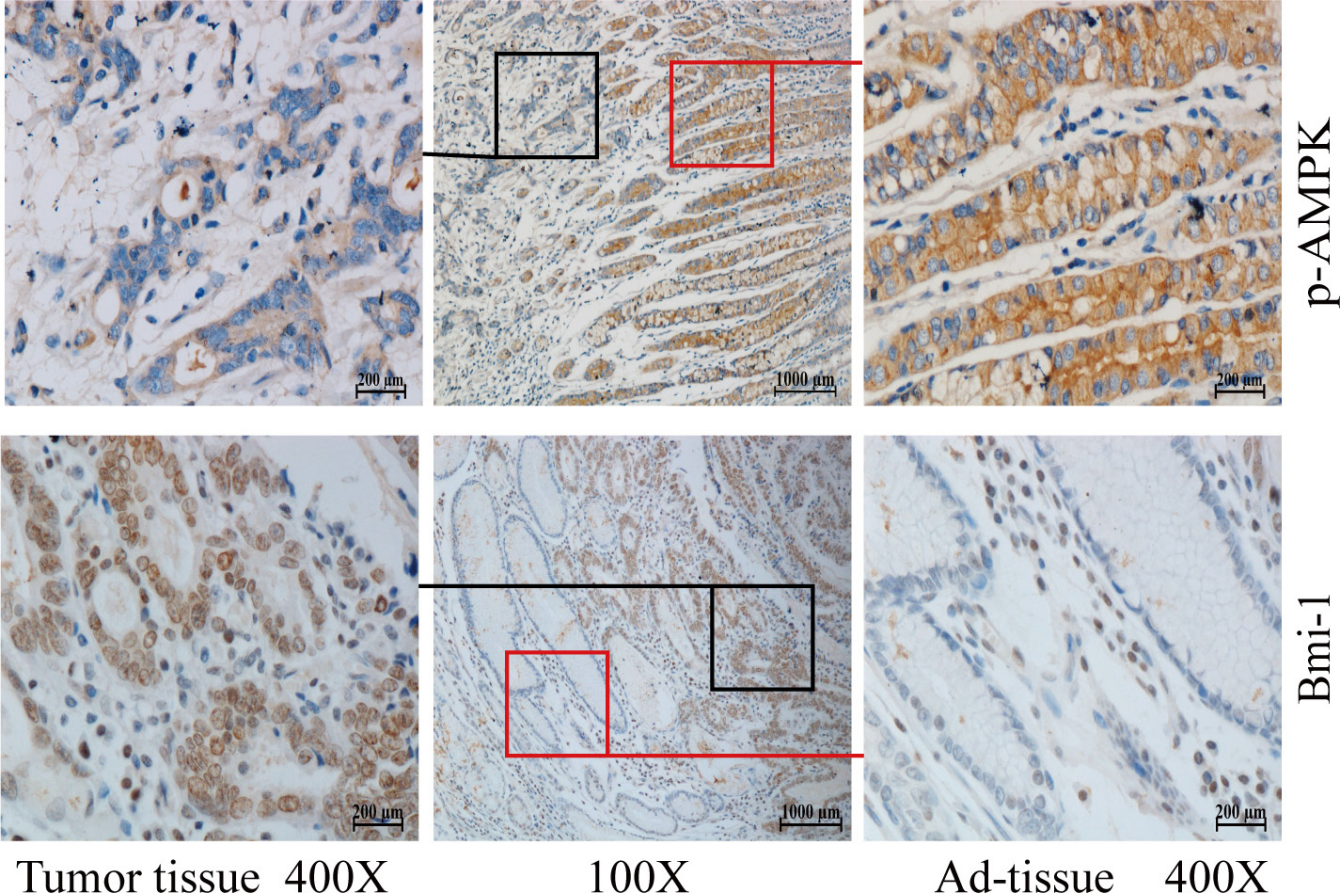
Phosphorylation of AMPK and Bmi-1 expression in gastric cancer Paraffin tissue blocks were sectioned from 66 gastric cancer specimens and slides immunohistochemically stained with anti-phosphorylated AMPK (T172) or Bmi-1 antibody and counterstained with hematoxylin. Representative images are shown for tumor and adjacent normal mucosae (Ad-tissue). The middle panel represents 100 x magnitude, and right (Ad-tissue) and left (tumor) panels are 400 x magnitude amplified from the islets indicated in the middle panel.

**Figure 2:**
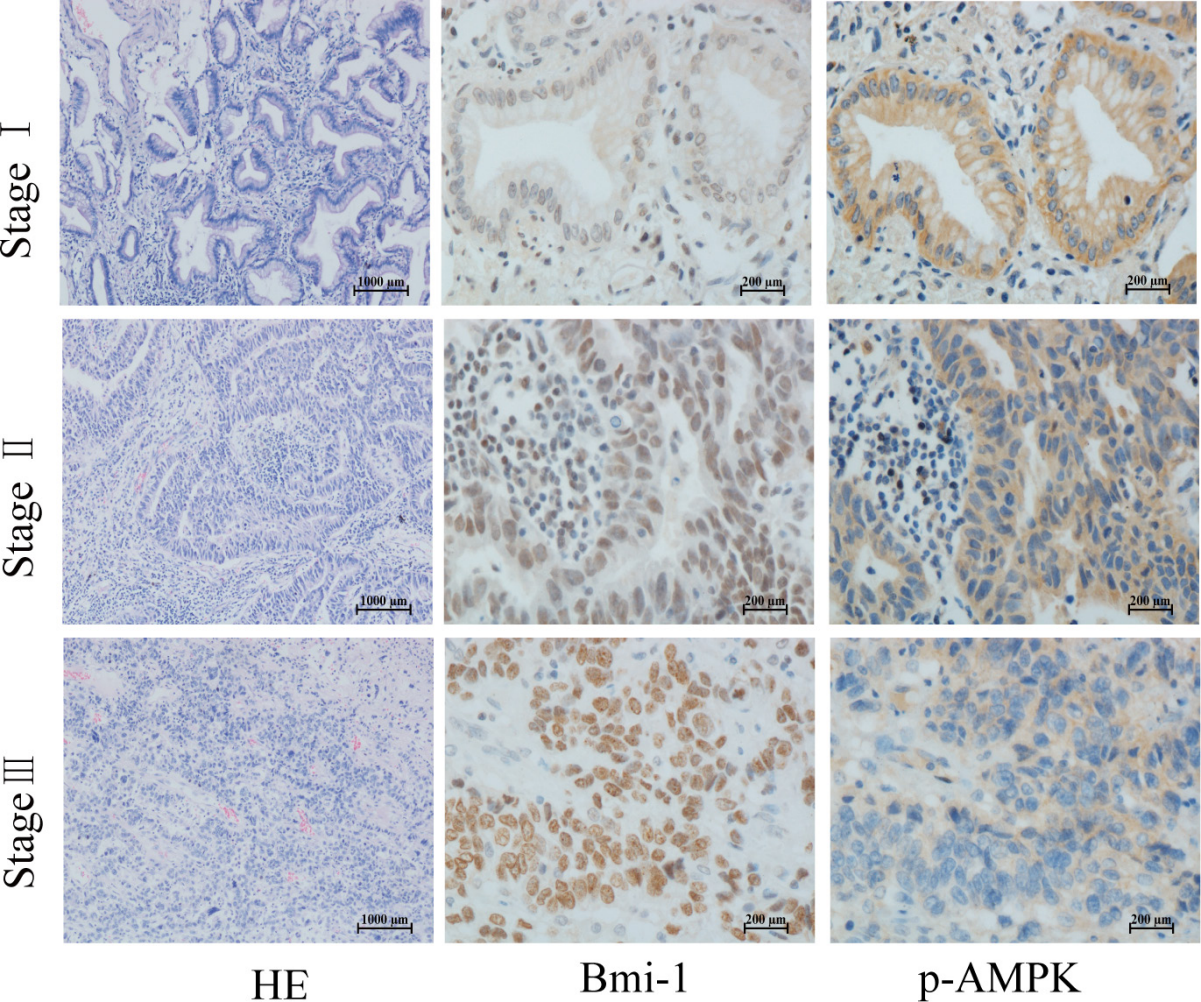
Phosphorylation of AMPK and Bmi-1 expression in lung cancer Paraffin tissue blocks were sectioned from 65 lung adenocarcinoma specimens at different pathological stages and stained with antibodies as described for Figure 1, and counterstained with hematoxylin and eosin (HE). Representative images are shown.

To explore the correlation between AMPK and Bmi-1, we collected 66 paraffin-embedded gastric cancer specimens and 65 lung adenocarcinoma specimens from the Department of Pathology, the First Affiliated Hospital of Nanchang University. The specimens were obtained from patients under the consent who underwent surgical resection. The specimens were sectioned and examined by immunohistochemical (IHC) staining with antibodies against p-AMPK (T172), a parameter of AMPK activation, and Bmi-1. Figures 1 and 2 showed representatives of IHC images. While phospho-AMPK was localized in the cytoplasm, Bmi-1 protein was present in the nucleus. In gastric cancer, the signal of p-AMPK was lower in tumor (*p* < 0.001) and Bmi-1 expression higher in tumor (*p* < 0.001) than that of adjacent normal tissues (Table 1, Figure 1). Similarly, the expression of Bmi-1 progressively increased while p-AMPK was decreased along with increases in pathological grade of lung adenocarcinomas; the order of intensity of p-AMPK (T172) appeared to be stageI > stage II > stage III (spearman correlation = −0.397, *p* < 0.05), while Bmi-1 expression was stage I < stage II < stage III (spearman correlation = 0.389, *p* < 0.05) (Table 2, Figure 2). As in the lung cancer tissue slides, we could not find adjacent normal tissue, we could not compare p-AMPK between normal and cancer cells in this study.

**Table 1:** Differential expression of p-AMPK and Bmi-1 proteins in gastric cancer tissues

*n* = 66	p-AMPK		*p* value	Bmi-1		*p* value
	Tumor	Ad-tissue		Tumor	Ad-tissue	
−	19	4	< 0.001	10	19	< 0.001
+	37	11		21	31	
++	8	33		12	14	
+++	2	18		23	2	

**Table 2:** The Bmi-1 and p-AMPK expression in different stages of lung cancer

stage	Total	p-AMPK				*p* value	Bmi-1				*p* value
	(*n* = 65)	−	+	++	+++		−	+	++	+++	
I	22	4	5	10	3	< 0.05	3	12	6	1	< 0.05
II	27	5	10	9	3		1	9	11	6	
III	16	9	6	1	0		0	5	4	7	

### Negative regulation of Bmi-1 expression by LKB1/AMPK

**Figure 3:**
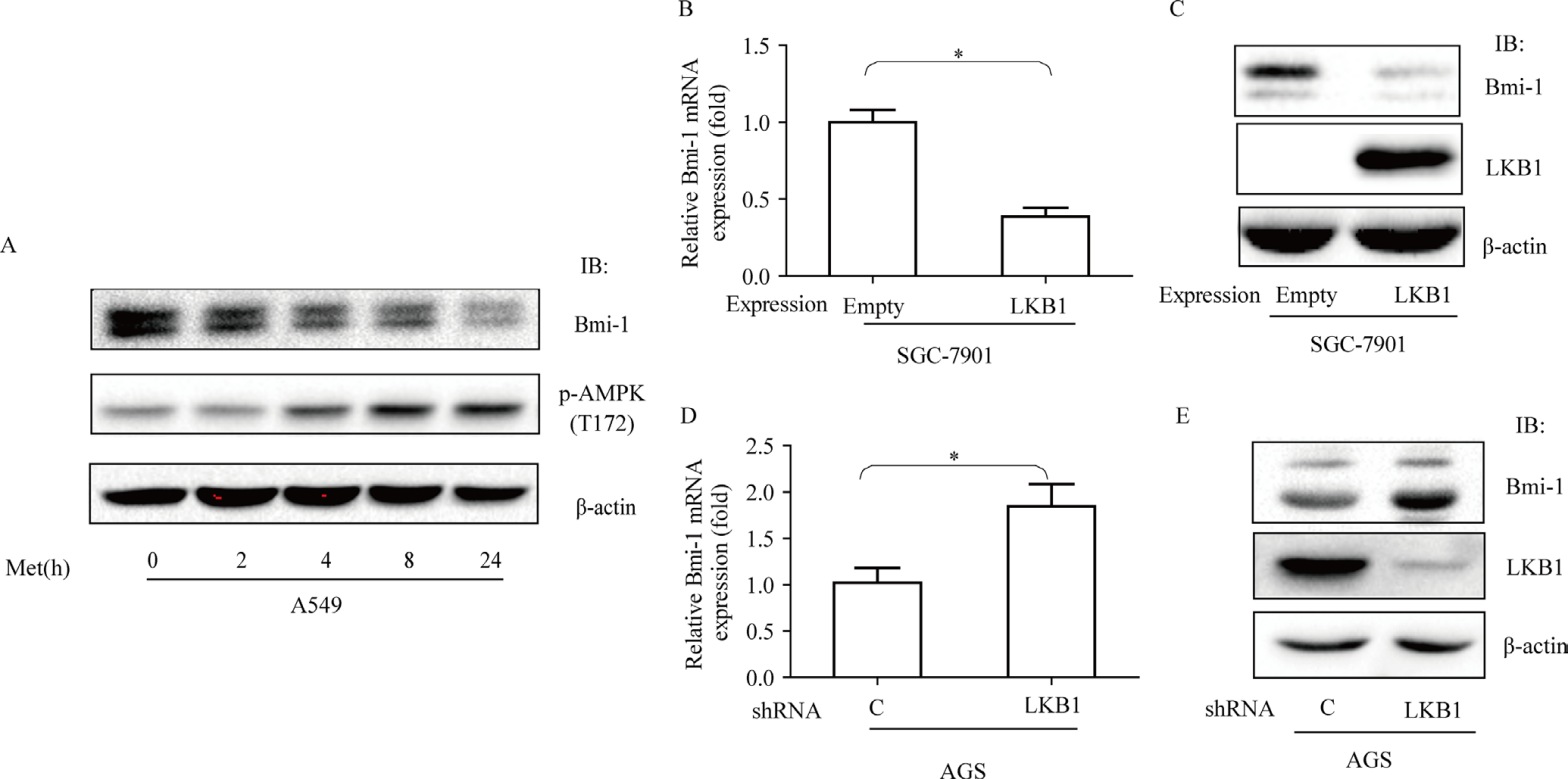
Effects of metformin and LKB1 on regulation of Bmi-1 expression (**A**) A549 cells were treated with metformin (10 mM) for different times and cell extracts blotted with antibodies, as indicated. (**B** & **C**) LKB1 was expressed in SGC-7901 cells, (**D** & **E**) LKB1 was silenced in AGS cells by shRNA. qRT-PCR for Bmi-1 mRNA was performed (B., D.) or protein was blotted with antibodies (C., E.), as indicated. Graphs represent mean ± SD (*n* = 3). Student *t* test was performed, *p < 0.05.

We assessed whether activation of AMPK exerts a causative effect on downregulation of Bmi-1 in cancer cells. First, we treated A549 cells, a lung adenocarcinoma cell line, with metformin and examined the expression of Bmi-1. We found that metformin increased phosphorylation of AMPK at T172 and simultaneously reduced the abundance of Bmi-1 (Figure 3A). Next, we made stable cells by ectopic expression of LKB1 in SGC-7901 cells, gastric cancer cell line in which endogenous LKB1 was very low and by infection of LKB1 shRNA in AGS, another gastric cancer cell line, and then measured mRNA and protein of Bmi-1. Our results revealed that ectopic expression of LKB1 diminished the levels of Bmi-1 mRNA and protein, while silencing LKB1 caused an opposite change of both (Figure 3B–3E).

**Table 3:** miRNA expression in LITAF-silencing A549 cells

Downregulation	fold	*p* value
hsa-miR-15a	6.1893246	0.02478829
hsa-miR-194	5.4402036	0.04578442
hsa-miR-128	4.1604925	0.01780326
hsa-miR-192	3.6139035	0.00870038

### The role of LITAF in regulation of Bmi-1

**Figure 4:**
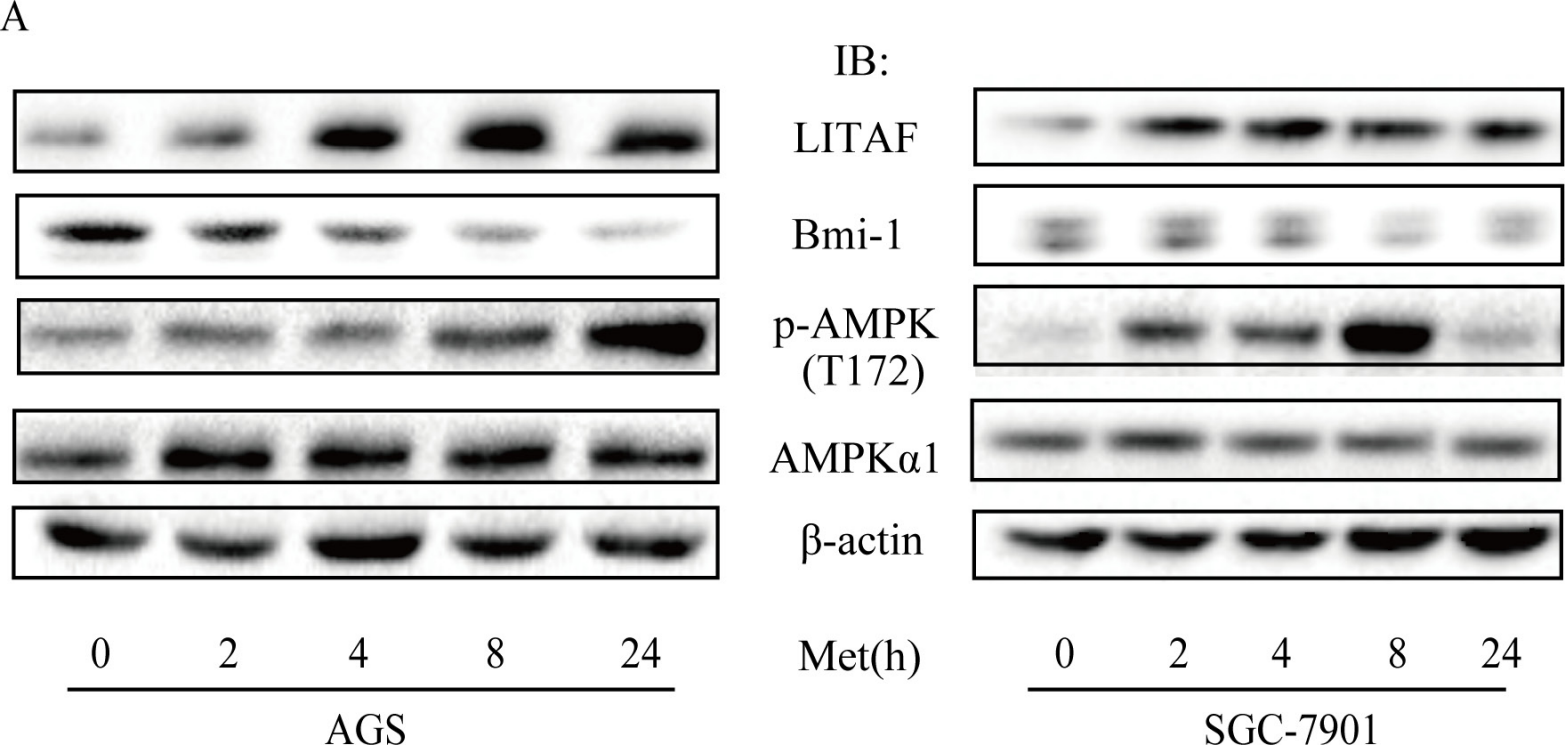
Regulation of LITAF and Bmi-1 by metformin AGS cells (**A**) and SGC-7901 cells (**B**) were treated with metformin (10 mM) for different times and cell extracts (20 μg) were blotted with antibodies, as indicated.

**Figure 5:**
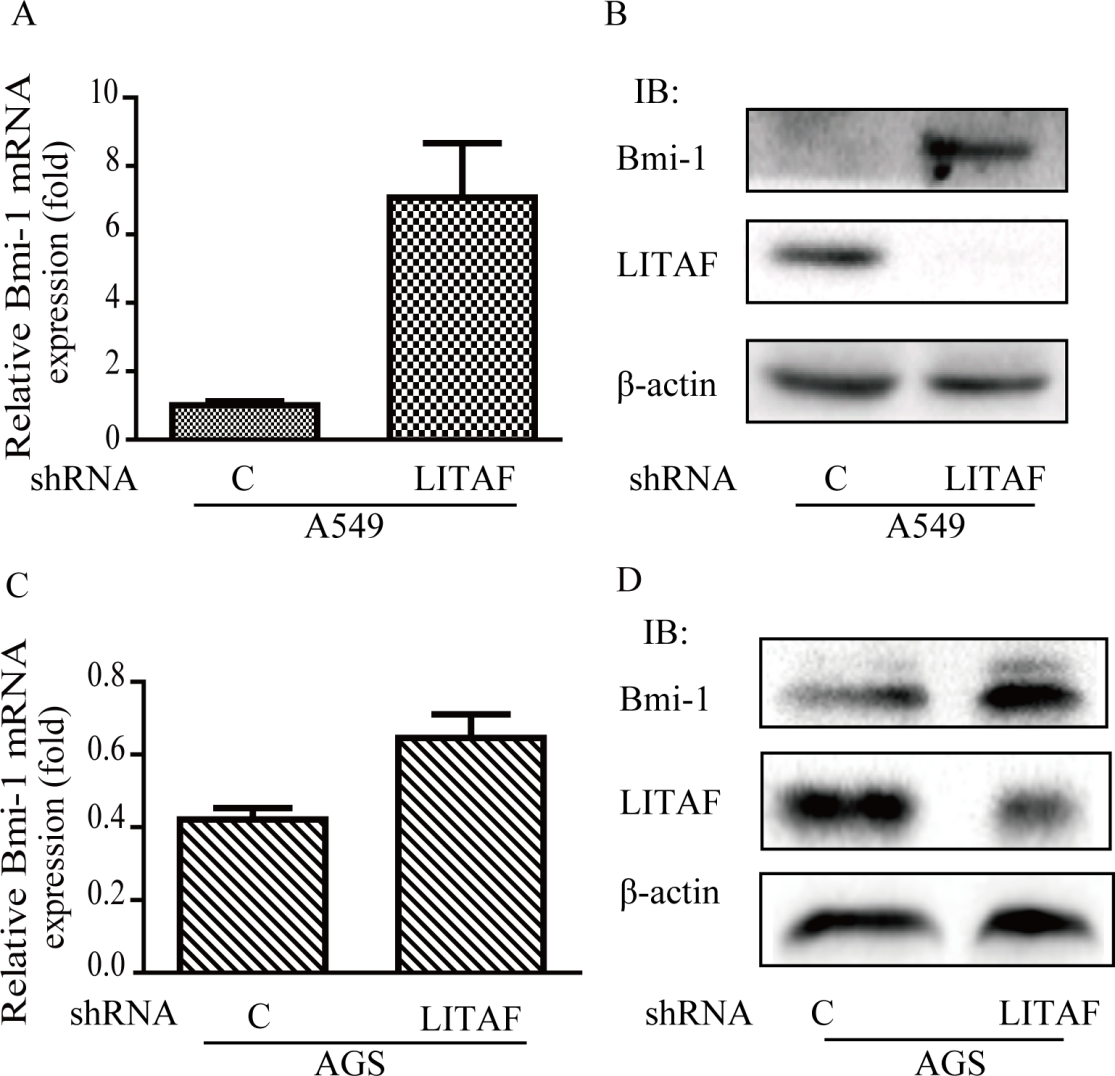
Induction of Bmi-1 by LITAF knockdown LITAF was silenced in A549 and AGS cells by shRNA vs. scrambled shRNA as a control. (**A** & **C**) Total RNA was prepared and subjected to qRT-PCR analysis for Bmi-1 expression. (**B** & **D**) Cell extracts were blotted with antibodies as indicated.

**Figure 6:**
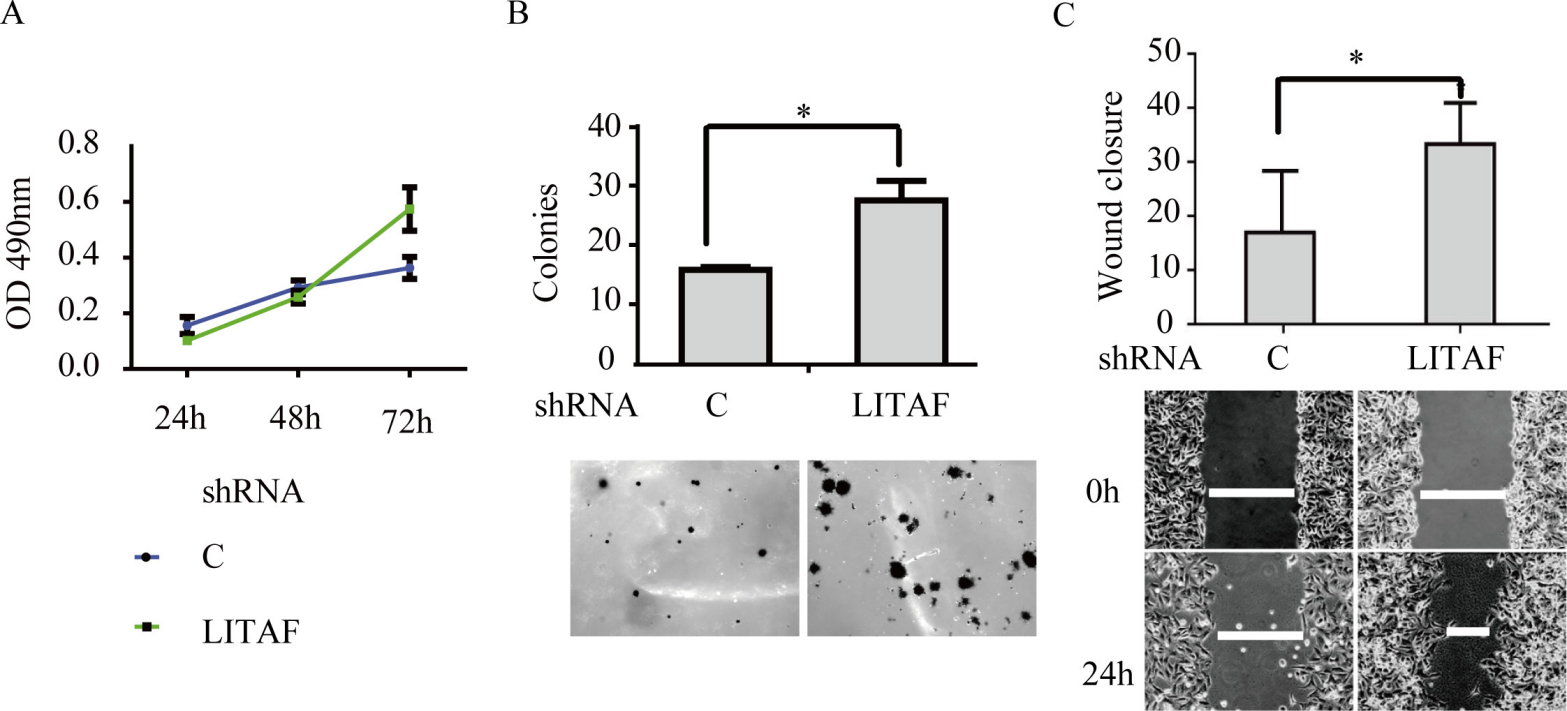
The effect of LITAF knockdown on cellular behavior of A549 cells (**A**) MTT assay. The cells were subjected to MTT assay for up to 72 h. (**B**) Colony formation assay. The cells were assayed for anchorage-independently growth on soft agar. (**C**) Wound-healing assay. The graphs represent quantitative data. Averages of a triplicate experiment were plotted (mean ± SD, *n* = 3), student *t* test, *p < 0.05.

To identify components downstream of AMPK in regulation of Bmi-1, we assessed if LITAF had a role. We first examined if the expression of LITAF and Bmi-1 was altered by metformin treatment. Our results showed that metformin upregulated LITAF and downregulated Bmi-1 in SGC-7901 and AGC cells (Figure 4) as well as A549 cells (data not shown). We then silenced LITAF by shRNA and examined the effect on Bmi-1 expression and cell behavior. As shown in Figure 5, knockdown of LITAF in A549 and AGS cells caused a marked increase in mRNA and protein levels of Bmi-1. This was associated with cell viability, colony formation, and migration of A549 cells (Figure 6). These results suggest that LITAF plays an inhibitory role in regulation of Bmi-1, which may contribute to inhibition of cancers cell progression.

### Role of miRNAs in LITAF-mediated regulation of Bmi-1

**Figure 7:**
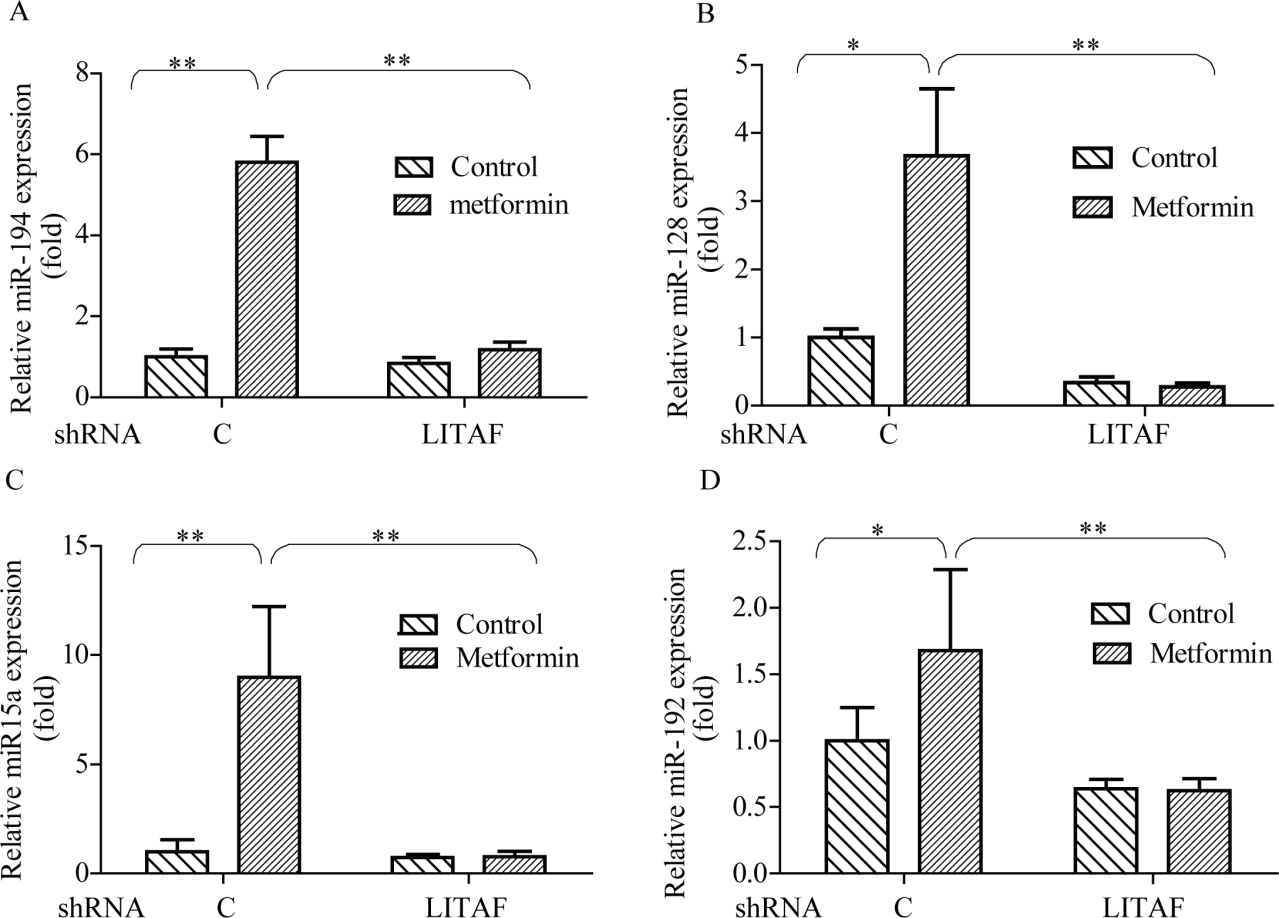
Effect of LITAF on miRNA regulation A549 cells with or without LITAF shRNA were treated with metformin (10 mM) for 24 hours and total RNA was isolated for qRT-PCR analysis of miRNAs, miR-194, miR-128, miR-15a, and miR-192. Each sample was amplified in triplicate and normalized to U6 expression. Results were evaluated by the comparative threshold cycle value method for relative quantification of gene expression. Graphs represent mean ± SD (*n* = 3), student *t* test,*p < 0.05, **p < 0.01.

**Figure 8:**
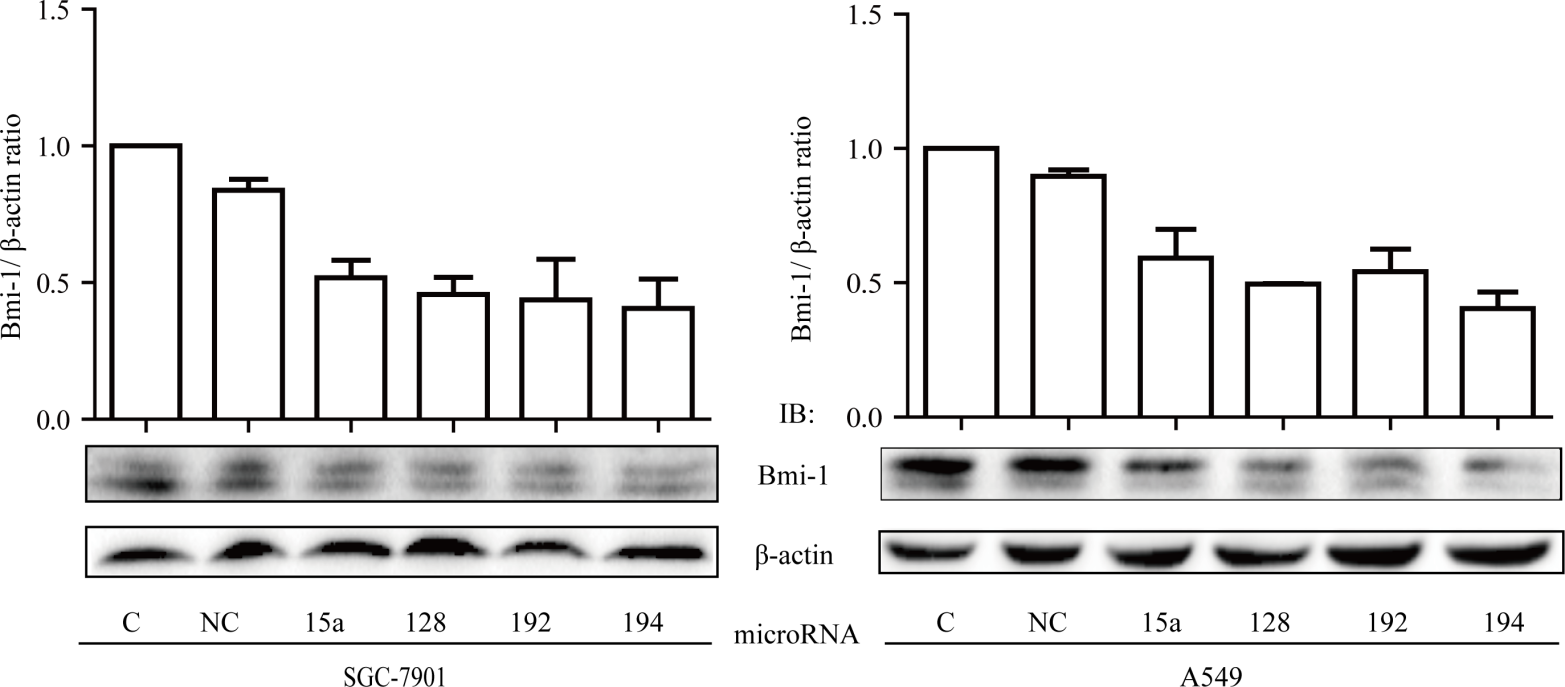
Inhibitory effect of miRNAs on Bmi-1 expression (**A**) SGC-7901 cells and A549 cells were transfect with miR-15a, miR-128, miR-192, and miR-194, respectively, as compared to control cells (C, non-transfected; NC, negative control mimic). Two days later, cells were harvested and extracts subjected to Western blot analysis of Bmi-1. Upper: graphs represent averages of scan densitometry units of each sample in a triplicate experiment that is normalized to β-actin. Lower: representatives of Western blots.

To decipher the mechanism by which LITAF regulates growth of cancer cells, we performed miRNA microarray analysis on the A549 cells where LITAF was silenced as compared with the control counterpart containing scrambled shRNA (Supplementary Table 1). We identified many miRNAs that were differentially expressed, among which miR-15a, miR-128, miR-192, and miR-194 were focused, as they were previously reported to regulate Bmi-1 [10–12, 26]. These microRNAs were increased by metformin, which was abolished by knockdown of LITAF (Figure 7). To further address if any of these miRNAs had a role in regulation of Bmi-1, we transfected each of them into SGC 7901 and A549 cells and then examined Bmi-1 expression. As shown in Figure 8, Bmi-1 was downregulated by transfection of each individual miRNAs.

## DISCUSSION

As a key factor in energy metabolism, AMPK is being widely accepted as a metabolic tumor suppressor [14]. Our previous study has identified LITAF as a downstream effector of AMPK in regulation of tumor cell growth; however, the mechanism by which it mediates the tumor suppressive function remains to be determined [23]. In the present study, we attempted to depict the linear relationship of AMPK, LITAF and Bmi-1 in cancer cells. We found that expression of Bmi-1 and p-AMPK inversely changed in gastric cancer tissues; p-AMPK level was reduced, whereas Bmi-1 expression was increased, as compared to adjacent tissues. Additionally, similar trend was found in lung adenocarcinoma specimens. Our findings of opposite changes in p-AMPK and Bmi-1 are interesting; however, further correlation analysis on these changes did not show significance, which was possibly reflected by insufficient number of samples used in this study. Nevertheless, the trend of changes prompted us to perform *in vitro* assays to delineate their relationship in cancer cells. Our results showed that AMPK activation increased the abundance of LITAF and concurrently reduced expression of Bmi-1, while knockdown of LITAF upregulated Bmi-1 and promoted aggressive behaviors of cancer cells. Furthermore, metformin activation upregulated miR-15a, miR-128, miR-192, and miR-194, which was abolished by knockdown of LITAF. Transfection of these individual miRNAs diminished the expression of Bmi-l. Therefore, our data for the first time indicate a regulatory axis of AMPK-LITAF-miRNA-Bmi-1.

Thus far, only a few reports have suggested that LITAF is involved in tumorigenesis. For example, LITAF is silenced by homologous deletion in primary mediastinal lymphoma and by promoter hypermethylation in germinal center lymphoma [27]. This work does not clearly delineate whether silencing of LITAF in these lymphomas is a coincident or cause-effect event. However, several studies suggest that LITAF may have a tumor suppressive role. First, LITAF could be downregulated by oncogenic protein BCL6 and ectopic expression of LITAF elicits autophagy [25]. Second, transfection of LITAF induces apoptosis of acute leukemia cells [24]. Third, we have previously shown that knockdown of LITAF promotes proliferation and migration of prostate cancer cells [23]. One of the underlying mechanisms is the induction of TNFSF15, an important negative regulator of tumor angiogenesis. In the current study, we extended our study to other cancer cells and confirmed the inhibitory effect of LITAF on malignant behaviors in A549 cancer cells. Furthermore, we identified Bmi-1 as another downstream target of LITAF. It is possible the tumor suppressive function of LITAF is mediated by its transcriptional activity [23] and its effect on autophagy [28].

Previous studies have demonstrated that dysregulated expression of Bmi-1 contributes to tumor cell self-renewal and EMT, critical steps for tumor progression and metastasis [29, 30]. It has been shown that Bmi-1 is regulated by miRNAs; for instance, miR-15a is able to bind to 3′-UTR of Bmi-1 mRNA to inhibit translation [10], miR-128 can inhibit Bmi-1 expression in drug-resistant cancer cells [31], and miR-194 blocks cancer cell EMT through inhibition of Bmi-1 expression [12]. All these miRNAs are known to be tumor-suppressing factors [10, 12, 31, 26].

In the present study, the concentration of metformin used was 10 mM, which is far above the concentration (20–40 μM) achieved by routine oral administration in plasma of patients with type 2 diabetes [32]. It has been recognized that the concentration of metformin required for *in vitro* activation of AMPK is much higher than that *in vivo* [33]. A possible explanation is that metformin transporter varies on different cell types, which results in different kinetics in activation of AMPK, as observed differences in AGS and SGC-7901 cells (Figure 4). When the expression of the transporter is low, the dose of metformin or incubation time will need to increase for optimal activation of AMPK. In most *in vitro* study, the incubation time with metformin is shorter than *in vivo* experiments to reach steady state concentration in plasma.

**Figure 9:**
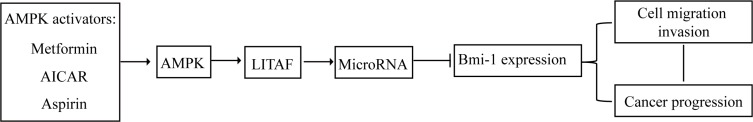
Model about down-regulation of Bmi-1 by AMPK and LITAF The diagram depicts the linear relationship of AMPK-LITAF-mRNA-Bmi-1-cancer progression.

Our present study showed that metformin increases the expression of these miRNA, which is abrogated by silencing LITAF. Therefore, our study for the first time demonstrates that Bmi-1 is downregulated by AMPK and LITAF, an event possibly occurring through upregulation of miRNAs (Figure 9). In sum, previous publications by us and others support that LITAF functions as a tumor suppressor. We have shown that LITAF acts as transcriptional factor to activate transcription of TNFSF15, so as to inhibit tumor angiogenesis [23]. Our present study revealed that LITAF upregulates a set of miRNAs. It is our future work to elucidate the mechanism underlying this regulation.

## MATERIALS AND METHODS

### Ethical statement and tissue specimens

The study entailing human gastric and lung cancer specimens was approved by the ethical committee of The First Hospital of Nanchang University (Nanchang, China) (Ethical Trial for Medical Research, 2014(025)). Tissue specimens were collected from surgical resection and deposited to pathological specimen library of The First Hospital of Nanchang University (Nanchang, China).

In this study, samples from 66 patients with gastric cancer were recruited, including 51 males and 15 females, with the age ranging from 34 to 79 years and the median age of 56.5 years. Cancer and paired adjacent tissues were obtained. In gastric cancer, differences in Bmi-1 and p-AMPK were compared between gastric cancer and adjacent tissue.

Study of lung cancer recruited samples of 65 patients with adenocarcinoma at different stages, including 22 cases of stage I, 27 cases of stage II, 16 cases of stage III. The patients include 36 males and 29 females, at the age from 35 to 80 years with the median age of 58.2 years. Pathological grading was made according to Barletta et al. [34]. Stage I is well differentiated, Stage II moderately differentiated, and Stage III poorly differentiated.

### Reagents

Antibodies against phosphorylated α (p-AMPKα Thr172) and non-phosphorylated AMPKα were purchased from Cell Signaling Technology (Danvers, MA, USA); Antibody against Bmi-1 was from Abcam (Cambridge, MA, USA); Antibodies against LKB1 and β-actin were from Santa Cruz Biotechnology (Santa Cruz, CA, USA). Anti-LITAF antibody was from Abnova Corporation (Taiwan, China). Diaminobenzidine (DAB) was from Dako (Carpinteria, CA, USA). Enzyme Antibody Conjugates were from ZSGB-BIO (Beijing, China). Metformin and 3-(4,5-dimethylthiazol-2-yl)-2,5-diphenyltetrazolium bromide (MTT) were from Sigma-Aldrich (St Louise, MO, USA). Trizol reagent, FastQuant RT Kit, and Quant SYBR Green PCR kit were from TIANGEN Biotech (Beijing, China). Lipfectamine 2000 was from Life Technologies (Grand Island, NY). miR-15a mimic, miR-128 mimic, miR-192 mimic, miR-194 mimic, and negative control mimic were purchased from Ribobio (Guangzhou, China). SuperSignal West Pico Chemiluminescence kit was from Thermo-Fisher scientific (Waltham, MA, USA).

### Immunohistochemistry

Immunohistochemistry was performed using a ZSGB-bio kit (ZSGB-BIO, Beijing, China) according to the manufacturer's instructions. Briefly, paraffin-embedded tissue blocks were sectioned to 5 microns. The tissue slides were deparaffinized and rehydrated into water and then subjected to treatment with citric acid buffer (pH 6.0, 10 mM) using standard microwave-based antigen retrieval method. The slides were blocked, incubated with antibodies against p-AMPK (Thr-172) (CST 2535L, 1:50) and Bmi-1(abcam 14389, 1:200) at 4°C overnight. The samples were then washed and incubated again with a secondary antibody against rabbit HRP-conjugated IgG. The reaction was developed using 3,3′-Diaminobenzidine (DAB) and counterstained with hematoxylin. The immunostained tissue sections were evaluated semi-quantitatively according to the immunoreactive scores (IRS) by German semi-quantitative statistical methods. Briefly, slices were evaluated by 2 pathologists independently under 200 x and 400 x optical microscope using positive staining intensity and percentage of positive staining. Positive staining intensity rating: no staining scores 0 points; light yellow scores 1; yellow scores 2; dark yellow (brown) scores 3. Scores for positive stained cells: if the number of positive glandular epithelial cells of the total number of glandular epithelial cells lies below 5%, counting 0 point; or over 5% and below 25% counting 1; or over 25% and < 50% counting 2; or over 50% and < 75% counting 3; or over 75% counting 4. The staining intensity and number of stained cells were integrated to final scores; scale of 0 ∼ 2 was regarded as negative, 3 ∼ 5 as +, 6 ∼ 8 as + +, 9 ∼ 12 as +++ [35].

### Cell culture, virus infection, transfection and metformin treating

The lung adenocarcinoma A549, human embryonic kidney HEK293T cells, human gastric carcinoma SGC-7901 cells and AGS cells were cultured in DMEM (Gibco, Life Technologies) supplemented with 10% fetal bovine serum in a 5% CO_2_ and 37°C incubator. Whenever needed, cells were treated with metformin dissolved in PBS at 10 mM or PBS vehicle for different periods of time, as indicated in the figure legends [36, 37].

Retroviral plasmids for LITAF shRNA and scrambled shRNA were purchased from Open Biosystems (Huntsville, AL, USA). Retrovirus for LITAF shRNA and lentivirus for LKB1 shRNA and LKB1 were packaged in HEK293T. The virus supernatant was infected into cells as noted in the results and Figure Legend. Two days after infection, the cells were selected with puromycin.

Cells were transfected with either miR-15a mimic, miR-128 mimic, miR-192 mimic, miR-194 mimic or negative control mimic at a final concentration of 50 nM using Lipofectamine 2000 according to manufacturer's protocol.

### Western blot

Total cellular protein was extracted in a lysis buffer (25 mM Tris-HCl, pH 8.0, 100 mM NaCl, 1 mM EDTA, 1 mM EGTA, 1 mM Na_3_VO_4_ and 25 mM β-glycerol-phosphate, 1 mM DTT, 1% NP-40, and protease inhibitors) on ice for 30 minutes and centrifuged at 14,000 × g and 4°C for 20 min. Protein samples were boiled in 1×SDS loading buffer, separated by sodium dodecyl sulfate-polyacrylamide gel electrophoresis (SDS-PAGE) and then transferred onto nitrocellulose membrane (GE Healthcare, Munich, Germany) using 200 mA power for 2 h. After blocking in 5% non-fat milk for 1 hour, membranes were incubated with primary antibodies at 4°C overnight. The next day, membranes were incubated with second antibodies. Signal was developed using the SuperSignal West Pico Chemiluminescence kit.

### qRT-PCR

Total RNA from cells was isolated using a Trizol reagent, and reversely transcribed into cDNA using a FastQuant RT Kit according to the manufacturers' instructions. qPCR analysis of gene expression was conducted using the Quant SYBR Green PCR kit, as described by protocols of the manufacturer. The primers were listed in Table 4.

**Table 4:** Primers used in Q-PCR

	Primer sequences (5′-3′)
hsa-miR-15a	RT-primer-5′-GTCGTATCCAGTGCAGGGTCCGAGGTATTCGCACTGGATACGACCACAAAC-3′
	Forward: GCGGCTAGCAGCACATAATGG Reverse: GTCGTATCCAGTGCAGGGTCC
hsa-miR-128	RT-primer: 5′-GTCGTATCCAGTGCAGGGTCCGAGGTATTCGCACTGGATACGACAAAGAG-3′
	PCR primer: Forward: TCCGATCACAGTGAACCGGT Reverse: GTGCAGGGTCCGAGGT
hsa-miR-192	RT-primer: 5′-GCTGTCAACGATACGCTACGTAACGGCATGACAGTGTTTTTTTTTTTTTTTTTTTTTTTTA-3′
	PCR primer: Forward: CTGACCTATGAATTGACAGCCA Reverse: GCTGTCAACGATACGCTACGT
hsa-miR-194	RT-primer: 5′-GCTGTCAACGATACGCTACGTAACGGCATGACAGTGTTTTTTTTTTTTTTTTTTTTTTTTG-3′
hsa-miR-194	RT-primer: 5′-GCTGTCAACGATACGCTACGTAACGGCATGACAGTGTTTTTTTTTTTTTTTTTTTTTTTTG-3′
U6	Forward: CGCTTCGGCAGCACATATAC Reverse: TTCACGAATTTGCGTGTCAT
Bmi-1	Forward: GCCTTCTCTGCTATGTCTGAA Reverse: CTGATGAACACACACCAACTT
GAPDH	Forward: CAGGGCTGCTTTTAACTCTGGT Reverse: GATTTTGGAGGGATCTCGCT

### MTT assay

Briefly, cells were seeded into 96-well plates at a density of 1500 cells/well and cultured for up to 72 h. MTT solution was then added into cell culture and incubated for additional 4 h. After that, the cell culture medium was replaced by addition of 150 μl dimethyl sulfoxide (DMSO), and optical density was measured at absorbance rate of 490 nm using a SpectraMax M Series Multi-Mode Microplate Reader (Molecular Devices, Sunnyvale, CA, USA). Cell viability was then plotted in graph.

### Wound healing assay

In brief, cells were seeded into 6-well plates at density of 5×10^5^ cells/well and grew to 100% confluence and then a scratch was made across the monolayer using a pipette tip. The cell culture plates were washed briefly with phosphate buffered saline (PBS) for 3 times and added fresh DMEM without FBS and further incubated for 24 h. Images of the cells were captured at the time of scratch and the end of experiment. The distance of cell wound healing was measured and migration rate calculated according to equation: (R_0h_–R_24h_/R_0h_).

### Soft-agar colony formation assay

Soft-agar colony formation assay was performed to determine the clonegenic capability of tumor cells in semisolid medium. Briefly, 5 × 10^5^ cells were mixed in 0.5% soft-agar and plated on top of 1 ml of 1% agar bed prepared in complete culture medium in six-well plates. After the soft-agar was solidified, 1.5 ml DMEM with 15% FBS were added into each well. The plates were incubated for 2 to 3 weeks at 37°C in a humidified atmosphere containing 5% CO_2_. Cell colonies were then visualized by staining with MTT and counted.

### miRNA Array

All procedures were performed by Boston University Microarray and Sequencing Resource Core Facility, as described in FlashTag^™^ Biotin RNA Labeling Kit for Affymetrix miRNA Arrays protocol (Genisphere Inc., Hatfield, PA, current version available at http://media.affymetrix.com/support/downloads/manuals/flashtag_user_guide.pdf). Briefly, total RNA was isolated using QIAGEN's miRNeasy kit (Qiagen, Valencia, CA) and the sample integrity was verified using RNA 6000 Nano Assay RNA chips run in Agilent 2100 Bioanalyzer (Agilent Technologies, Palo Alto, CA). The RNA was then labeled with FlashTag HSR kit (Genisphere Inc., Hatfield, PA) according to the manufacturer's protocol. The labeled RNA was hybridized to the miRNA Galaxy arrays (Affymetrix, Santa Clara, CA) for 16 hours in GeneChip Hybridization oven 640 at 48°C with rotation (60 rpm). The hybridized samples were washed and stained using Affymetrix fluidics station 450. The first stain with streptavidin-R-phycoerythrin (SAPE) was followed by signal amplification using a biotinilated goat anti-streptavidin antibody and another SAPE staining (Hybridization, Washing and Sataining Kit, Affymetrix, Santa Clara, CA). Microarrays were immediately scanned using Affymetrix GeneArray Scanner 3000 7G Plus (Affymetrix, Santa Clara, CA). Affymetrix miRNA QC Tool software http://www.affymetrix.com/estore/browse/products.jsp?categoryIdClicked=&productId=131558) was used for summarization, normalization and quality control of the resulting CEL files.

### Statistical analysis

Chi-square test was used to compare the difference of Immunohistochemistry results and spearman analysis to examine correlations. Quantitative data between groups were expressed as mean ± standard deviation (SD) and analyzed by using *t*-test. A *p* value less than or equal to 0.05 was considered statistically significant.

## SUPPLEMENTARY MATERIALS TABLE



## Abbreviations

AMPK: Adenosine 5′-monophosphate (AMP)-activated protein kinase
LKB1: Liver kinase B1
LITAF: Lipopolysaccharide-induced TNFa factor
Bmi-1: B-lymphoma Moloney murine leukemia virus insertion region-1
EMT: Epithelial to mesenchymal transition
LPS: Lipopolysaccharides
SIMPLE: Small integral membrane protein of the lysosome/late endosome
BCL6: B-cell lymphoma 6 protein
TNF-a: Tumor necrosis factor alpha
TNFSF15: TNF superfamily member 15

## References

[1] Cao R, Tsukada Y-i, Zhang Y (2005). Role of Bmi-1 and Ring1A in H2A Ubiquitylation and Hox Gene Silencing. Molecular Cell.

[2] Hu J, Liu YL, Piao SL, Yang DD, Yang YM, Cai L (2012). Expression patterns of USP22 and potential targets BMI-1, PTEN, p-AKT in non-small-cell lung cancer. Lung Cancer.

[3] Huang KH, Liu JH, Li XX, Song LB, Zeng MS (2007). Association of Bmi-1 mRNA expression with differentiation, metastasis and prognosis of gastric carcinoma. [Article in Chinese]. Nan Fang Yi Ke Da Xue Xue Bao.

[4] Guo BH, Feng Y, Zhang R, Xu LH, Li MZ, Kung HF, Song LB, Zeng MS (2011). Bmi-1 promotes invasion and metastasis, and its elevated expression is correlated with an advanced stage of breast cancer. Mol Cancer.

[5] Park IK, Morrison SJ, Clarke MF (2004). Bmi1, stem cells, and senescence regulation. J Clin Invest.

[6] Saudy NS, Fawzy IM, Azmy E, Goda EF, Eneen A, Abdul Salam EM (2014). BMI1 gene expression in myeloid leukemias and its impact on prognosis. Blood Cells, Molecules, and Diseases.

[7] Renkonen S, Hayry V, Heikkila P, Leivo I, Haglund C, Makitie AA, Hagstrom J (2011). Stem cell-related proteins C-KIT, C-MYC and BMI-1 in juvenile nasopharyngeal angiofibroma—do they have a role?. Virchows Arch.

[8] Cao L, Bombard J, Cintron K, Sheedy J, Weetall ML, Davis TW (2011). BMI1 as a novel target for drug discovery in cancer. J Cell Biochem.

[9] Nacerddine K, Beaudry JB, Ginjala V, Westerman B, Mattiroli F, Song JY, van der Poel H, Ponz OB, Pritchard C, Cornelissen-Steijger P, Zevenhoven J, Tanger E, Sixma TK (2012). Akt-mediated phosphorylation of Bmi1 modulates its oncogenic potential, E3 ligase activity, and DNA damage repair activity in mouse prostate cancer. J Clin Invest.

[10] Bhattacharya R, Nicoloso M, Arvizo R, Wang E, Cortez A, Rossi S, Calin GA, Mukherjee P (2009). MiR-15a and MiR-16 control Bmi-1 expression in ovarian cancer. Cancer Res.

[11] Godlewski J, Nowicki MO, Bronisz A, Williams S, Otsuki A, Nuovo G, Raychaudhury A, Newton HB, Chiocca EA, Lawler S (2008). Targeting of the Bmi-1 oncogene/stem cell renewal factor by microRNA-128 inhibits glioma proliferation and self-renewal. Cancer Res.

[12] Dong P, Kaneuchi M, Watari H, Hamada J, Sudo S, Ju J, Sakuragi N (2011). MicroRNA-194 inhibits epithelial to mesenchymal transition of endometrial cancer cells by targeting oncogene BMI-1. Mol Cancer.

[13] Blagosklonny MV, Venkataraman S, Alimova I, Fan R, Harris P, Foreman N, Vibhakar R (2010). MicroRNA 128a Increases Intracellular ROS Level by Targeting Bmi-1 and Inhibits Medulloblastoma Cancer Cell Growth by Promoting Senescence. PLoS One.

[14] Luo Z, Zang M, Guo W (2010). AMPK as a metabolic tumor suppressor: control of metabolism and cell growth. Future Oncol.

[15] Hirsch HA, Iliopoulos D, Struhl K (2013). Metformin inhibits the inflammatory response associated with cellular transformation and cancer stem cell growth. Proc Natl Acad Sci U S A.

[16] Hardie D, Alessi DR (2013). LKB1, AMPK and the cancer-metabolism link - ten years after. BMC Biology.

[17] Lacerda AF, Hartjes E, Brunetti CR (2014). LITAF mutations associated with Charcot-Marie-Tooth disease 1C show mislocalization from the late endosome/lysosome to the mitochondria. PLoS One.

[18] Myokai F, Takashiba S, Lebo R, Amar S (1999). A novel lipopolysaccharide-induced transcription factor regulating tumor necrosis factor alpha gene expression: molecular cloning, sequencing, characterization, and chromosomal assignment. Proc Natl Acad Sci U S A.

[19] Tang X, Marciano DL, Leeman SE, Amar S (2005). LPS induces the interaction of a transcription factor, LPS-induced TNF-alpha factor, and STAT6(B) with effects on multiple cytokines. Proc Natl Acad Sci U S A.

[20] Bennett CL, Shirk AJ, Huynh HM, Street VA, Nelis E, Van Maldergem L, De Jonghe P, Jordanova A, Guergueltcheva V, Tournev I, Van Den Bergh P, Seeman P, Mazanec R (2004). SIMPLE mutation in demyelinating neuropathy and distribution in sciatic nerve. Ann Neurol.

[21] Beauvais K, Furby A, Latour P (2006). Clinical, electrophysiological and molecular genetic studies in a family with X-linked dominant Charcot-Marie-Tooth neuropathy presenting a novel mutation in GJB1 Promoter and a rare polymorphism in LITAF/SIMPLE. Neuromuscul Disord.

[22] Latour P, Gonnaud PM, Ollagnon E, Chan V, Perelman S, Stojkovic T, Stoll C, Vial C, Ziegler F, Vandenberghe A, Maire I (2006). SIMPLE mutation analysis in dominant demyelinating Charcot-Marie-Tooth disease: three novel mutations. J Peripher Nerv Syst.

[23] Zhou J, Yang Z, Tsuji T, Gong J, Xie J, Chen C, Li W, Amar S, Luo Z (2011). LITAF and TNFSF15, two downstream targets of AMPK, exert inhibitory effects on tumor growth. Oncogene.

[24] Liu J, Xing H, Chen Y, Wang L, Wang D, Rao Q, Tang K, Tian Z, He K, Wang M, Wang J (2012). PIG7, transactivated by AML1, promotes apoptosis and differentiation of leukemia cells with AML1-ETO fusion gene. Leukemia.

[25] Bertolo C, Roa S, Sagardoy A, Mena-Varas M, Robles EF, Martinez-Ferrandis JI, Sagaert X, Tousseyn T, Orta A, Lossos IS, Amar S, Natkunam Y, Briones J (2013). LITAF, a BCL6 target gene, regulates autophagy in mature B-cell lymphomas. Br J Haematol.

[26] Khella HW, Bakhet M, Allo G, Jewett MA, Girgis AH, Latif A, Girgis H, Von Both I, Bjarnason GA, Yousef GM (2013). miR-192, miR-194 and miR-215: a convergent microRNA network suppressing tumor progression in renal cell carcinoma. Carcinogenesis.

[27] Mestre-Escorihuela C, Rubio-Moscardo F, Richter JA, Siebert R, Climent J, Fresquet V, Beltran E, Agirre X, Marugan I, Marin M, Rosenwald A, Sugimoto KJ, Wheat LM (2007). Homozygous deletions localize novel tumor suppressor genes in B-cell lymphomas. Blood.

[28] Lee SM, Olzmann JA, Chin LS, Li L (2011). Mutations associated with Charcot-Marie-Tooth disease cause SIMPLE protein mislocalization and degradation by the proteasome and aggresome-autophagy pathways. J Cell Sci.

[29] Wu KJ (2011). Direct activation of Bmi1 by Twist1: implications in cancer stemness, epithelial-mesenchymal transition, and clinical significance. Chang Gung Med J.

[30] Wu CY, Hung JJ, Wu KJ (2012). Linkage between Twist1 and Bmi1: molecular mechanism of cancer metastasis/stemness and clinical implications. Clin Exp Pharmacol Physiol.

[31] Zhu Y, Yu F, Jiao Y, Feng J, Tang W, Yao H, Gong C, Chen J, Su F, Zhang Y, Song E (2011). Reduced miR-128 in breast tumor-initiating cells induces chemotherapeutic resistance via Bmi-1 and ABCC5. Clin Cancer Res.

[32] He H, Ke R, Lin H, Ying Y, Liu D, Luo Z (2015). Metformin, an old drug, brings a new era to cancer therapy. Cancer J.

[33] Viollet B, Guigas B, Sanz Garcia N, Leclerc J, Foretz M, Andreelli F (2012). Cellular and molecular mechanisms of metformin: an overview. Clin Sci (Lond).

[34] Barletta JA, Yeap BY, Chirieac LR (2010). Prognostic significance of grading in lung adenocarcinoma. Cancer.

[35] Remmele W, Stegner HE (1987). Recommendation for uniform definition of an immunoreactive score (IRS) for immunohistochemical estrogen receptor detection (ER-ICA) in breast cancer tissue. [Article in German]. Pathologe.

[36] Cho K, Chung JY, Cho SK, Shin HW, Jang IJ, Park JW, Yu KS, Cho JY (2015). Antihyperglycemic mechanism of metformin occurs via the AMPK/LXRalpha/POMC pathway. Sci Rep.

[37] Storozhuk Y, Hopmans SN, Sanli T, Barron C, Tsiani E, Cutz JC, Pond G, Wright J, Singh G, Tsakiridis T (2013). Metformin inhibits growth and enhances radiation response of non-small cell lung cancer (NSCLC) through ATM and AMPK. Br J Cancer.

