# Supplementary material for: Negative regulation of Bmi-1 by AMPK and implication in cancer progression
Source: Oncotarget. 2015 Dec 23;7(5):6188–200. doi: 10.18632/oncotarget.6748 (PMC4868749; doi:10.18632/oncotarget.6748)
Supplement: Supplementary file 1 [file oncotarget-07-6188-s001.pdf]

Negative regulation of Bmi–1 by AMPK and implication in cancer progression

Supplementary Materials

Supplementary Table 1: miRNA array data from A549 cells

ProbeSet.Name	*p*.value	fdr	log2.fold (AvC)		A2	A3	C1	C2	C3
hsa-miR-15a_st	0.024788	0.994828	–2.63	1.437806	3.704959	1.823996	4.85454	5.459805	4.541762
hsa-miR-192-star_st	0.045784	0.994828	–2.44	2.032307	2.560051	1.46264	5.144847	5.359276	2.881857
hsa-miR-128_st	0.017803	0.994828	–2.06	1.384006	2.430769	1.738113	4.718656	3.246898	3.757597
hsa-miR-192_st	0.0087	0.994828	–1.85	7.18363	8.462399	7.718947	9.84241	9.462162	9.621078
hsa-miR-194_st	0.007307	0.994828	–1.80	8.864582	9.591827	8.474003	10.97284	10.85171	10.49905
hsa-miR-194-star_st	0.04583	0.994828	–1.76	4.00437	2.846673	2.007033	5.042256	4.758053	4.334044
hsa-miR-484_st	0.007601	0.994828	–1.26	1.274299	1.600556	1.310035	3.002953	2.217731	2.748047
hsa-miR-301a_st	0.018455	0.994828	–1.03	0.744912	0.295191	0.606011	2.036424	1.293329	1.398553
hsa-miR-212_st	0.045572	0.994828	–0.89	0.739753	0.754072	1.371226	2.208856	1.892919	1.422393
hsa-miR-30e-star_st	0.040258	0.994828	–0.88	0.391352	0.867356	1.191956	1.431435	1.6167	2.036958
hsa-miR-362-5p_st	0.011515	0.994828	–0.66	5.020244	4.878596	5.033367	5.898325	5.42274	5.579332
hsa-miR-337-5p_st	0.049044	0.994828	–0.58	0.386888	0.744541	0.59819	1.188515	1.452747	0.83132
hsa-miR-606_st	0.032753	0.994828	–0.58	0.490356	0.52694	0.569719	0.810105	1.086949	1.431238
U82_st	0.037751	0.994828	–0.51	1.174019	0.964189	1.261502	1.373505	1.70235	1.852069
hsa-miR-612_st	0.011198	0.994828	–0.48	0.437879	0.523785	0.584746	1.036914	0.804665	1.136239
hsa-miR-616_st	0.002359	0.695471	–0.47	0.627047	0.448707	0.662581	1.069976	1.060837	1.014783
hsa-miR-508-3p_st	0.022376	0.994828	–0.43	0.561207	0.695918	0.754645	1.228148	1.185026	0.894595
hsa-miR-297_st	0.041107	0.994828	–0.42	0.501454	0.398826	0.746777	0.951144	0.812858	1.149199

hsa-miR-557_st	0.010611	0.994828	–0.38	0.354361	0.31485	0.286899	0.854577	0.646493	0.587251
hsa-miR-548e_st	0.013164	0.994828	–0.36	0.498538	0.338089	0.515727	0.696119	0.916408	0.829179
hsa-miR-526a_st	0.034836	0.994828	–0.35	0.503401	0.568164	0.309158	0.806675	0.946944	0.6728
hsa-miR-105_st	0.003698	0.934473	–0.33	0.487395	0.547328	0.672627	0.917178	0.892529	0.901662
ENSG00000201810_st	0.043887	0.994828	–0.28	0.5459	0.747555	0.638791	0.886775	1.074678	0.815133
ENSG00000212148_x_st	0.008715	0.994828	–0.28	0.866048	1.016755	0.85788	1.138686	1.216303	1.220716
ENSG00000201705_st	0.02622	0.994828	–0.25	0.622594	0.585078	0.672245	0.920807	0.74551	0.975013
ENSG00000212395_x_st	0.008319	0.994828	–0.25	0.873805	0.724178	0.76811	1.004421	1.018437	1.085638
ENSG00000212411_st	0.03286	0.994828	–0.25	0.447687	0.652532	0.530203	0.88224	0.776867	0.713272
ENSG00000207274_x_st	0.048115	0.994828	–0.25	0.718157	0.469397	0.515731	0.741446	0.890048	0.811443
HBII-85-1_x_st	0.002073	0.695471	–0.23	0.836447	0.75487	0.842038	1.043436	1.013809	1.070474
hsa-miR-432-star_st	0.028743	0.994828	–0.21	0.584823	0.475629	0.552774	0.653842	0.837648	0.745354
ENSG00000207268_x_st	0.035438	0.994828	–0.20	0.606117	0.47497	0.63848	0.853395	0.748171	0.720542
ENSG00000199282_st	0.01611	0.994828	–0.20	0.624761	0.670903	0.698875	0.918231	0.774625	0.89297
HBII-52-32_x_st	0.004549	0.994828	–0.17	0.567173	0.661664	0.581302	0.773177	0.768736	0.777133
ENSG00000207471_x_st	0.014924	0.994828	–0.09	0.698493	0.667103	0.702484	0.777593	0.812858	0.747438
ENSG00000201393_st	0.016912	0.994828	0.07	0.85034	0.876962	0.898761	0.782316	0.823191	0.803898
ENSG00000206901_st	0.041497	0.994828	0.1	0.887164	0.954161	0.912169	0.865022	0.823465	0.774156
hsa-miR-373_st	0.025614	0.994828	0.11	0.530811	0.519049	0.43721	0.394258	0.399514	0.357443

ENSG00000206649_st	0.049156	0.994828	0.15	0.628738	0.77847	0.77111	0.606596	0.589911	0.534331
ENSG00000212273_x_st	0.048185	0.994828	0.15	0.940284	0.9384	0.808451	0.738195	0.80104	0.688495
ENSG00000202335_st	0.016191	0.994828	0.15	0.83673	0.879422	0.76522	0.699833	0.683055	0.633744
ENSG00000207516_st	0.04112	0.994828	0.17	0.766715	0.865447	0.713994	0.588029	0.683484	0.572023
hsa-miR-220c_st	0.040329	0.994828	0.17	0.628122	0.808733	0.786446	0.572283	0.5741	0.566483
ENSG00000212445_st	0.038039	0.994828	0.18	0.739753	0.837298	0.891171	0.562937	0.673554	0.688279
hsa-miR-196a-star_st	0.046161	0.994828	0.19	0.744912	0.89666	0.838239	0.596941	0.735016	0.572509
ENSG00000202434_st	0.031742	0.994828	0.21	0.742527	0.815127	0.886596	0.70421	0.552205	0.557217
U86_st	0.030325	0.994828	0.22	1.164756	1.154536	1.31915	1.07305	0.968254	0.948544
ACA29_st	0.040124	0.994828	0.24	0.792244	0.906014	0.819282	0.74206	0.509014	0.55999
hsa-miR-206_st	0.037895	0.994828	0.24	0.685991	0.945514	0.76715	0.544729	0.584001	0.55999
ENSG00000206947_x_st	0.037009	0.994828	0.25	0.843767	0.667581	0.935497	0.593878	0.5732	0.539047
ENSG00000200222_st	0.023107	0.994828	0.25	0.775825	0.896686	0.758433	0.655385	0.466878	0.55999
ENSG00000206869_x_st	0.02548	0.994828	0.26	0.750826	0.823329	0.656577	0.374057	0.553515	0.530029
ACA14b_st	0.010911	0.994828	0.27	0.972429	0.954459	1.042696	0.626837	0.81311	0.719729
ENSG00000201733_x_st	0.027266	0.994828	0.27	1.007614	0.892197	0.920914	0.810105	0.622845	0.575108
ENSG00000200294_st	0.001839	0.695471	0.28	0.69156	0.718827	0.682976	0.366093	0.489296	0.412523
hsa-miR-488_st	0.020189	0.994828	0.28	0.664974	0.821883	0.692714	0.40404	0.376248	0.55999
ENSG00000200753_st	0.029386	0.994828	0.28	0.752392	0.839864	0.720256	0.454272	0.636899	0.378322

ENSG00000212224_st	0.01236	0.994828	0.3	1.157339	1.018661	1.025119	0.779115	0.668013	0.849962
hsa-miR-301b_st	0.037505	0.994828	0.33	1.042846	0.970617	0.801437	0.456295	0.679511	0.702572
hsa-miR-525-3p_st	0.025569	0.994828	0.33	0.986813	1.083624	0.859785	0.76585	0.654821	0.529559
ENSG00000212397_st	0.041934	0.994828	0.35	1.172278	1.016755	1.384533	0.92261	0.757465	0.858415
hsa-let-7a-star_st	0.013136	0.994828	0.35	0.625188	0.751279	0.807016	0.280976	0.365689	0.493131
ACA67_st	0.001098	0.695471	0.38	1.188048	1.077846	1.182285	0.825435	0.74562	0.741379
hsa-miR-587_st	0.017234	0.994828	0.39	0.809393	0.695918	0.687318	0.345457	0.190256	0.50191
ENSG00000201863_x_st	0.027162	0.994828	0.39	0.726733	0.8565	0.909429	0.265663	0.618753	0.427765
ENSG00000202498_x_st	0.044521	0.994828	0.4	1.171086	0.715978	0.830362	0.546423	0.512861	0.444471
hsa-miR-92b_st	0.04549	0.994828	0.44	6.672776	6.562646	6.465828	6.401174	5.934834	6.059641
hsa-miR-633_st	0.045146	0.994828	0.44	0.997296	1.08575	0.614667	0.368	0.537696	0.473294
hsa-miR-888_st	0.023774	0.994828	0.45	0.660717	0.888844	0.938457	0.198618	0.511807	0.427301
hsa-miR-541_st	0.008499	0.994828	0.46	0.88017	0.914599	0.811017	0.464096	0.533835	0.236669
hsa-miR-607_st	0.029143	0.994828	0.51	0.744912	1.180935	1.170269	0.49558	0.453073	0.624242
HBII-85-26_x_st	0.005996	0.994828	0.57	1.243768	1.140675	1.337705	0.810105	0.691936	0.495245
hsa-miR-198_st	0.030629	0.994828	0.59	1.408067	1.397638	1.039735	0.49558	0.944436	0.646028
ENSG00000201660_st	0.001725	0.695471	0.62	1.334182	1.254642	1.489076	0.834379	0.694925	0.700248
hsa-miR-210_st	0.045887	0.994828	0.72	7.648463	7.309572	8.169192	6.921895	7.016421	7.024682
hsa-miR-27b-star_st	0.024861	0.994828	0.74	7.672674	8.00034	8.325807	7.292222	7.079647	7.406055

hsa-miR-885-5p_st	0.000972	0.695471	0.8	1.428763	1.544284	1.611914	0.831411	0.58549	0.777854
hsa-miR-181a-2-star_st	0.016395	0.994828	0.95	5.467124	6.067974	5.712282	4.960817	4.964092	4.474508

Note: miRNA from A549 cells, A1–A3, triplicates of LITAF shRNA; C1–C3. triplicates of control
